# Antagonistic neural networks underlying differentiated leadership roles

**DOI:** 10.3389/fnhum.2014.00114

**Published:** 2014-03-04

**Authors:** Richard E. Boyatzis, Kylie Rochford, Anthony I. Jack

**Affiliations:** ^1^Department of Cognitive Science, Case Western Reserve UniversityCleveland, OH, USA; ^2^Department of Organizational Behavior, Case Western Reserve UniversityCleveland, OH, USA

**Keywords:** leadership, roles, neural networks and behavior, DMN, TPN, anti-correlated networks, opposing domains hypothesis

## Abstract

The emergence of two distinct leadership roles, the task leader and the socio-emotional leader, has been documented in the leadership literature since the 1950s. Recent research in neuroscience suggests that the division between task-oriented and socio-emotional-oriented roles derives from a fundamental feature of our neurobiology: an antagonistic relationship between two large-scale cortical networks – the task-positive network (TPN) and the default mode network (DMN). Neural activity in TPN tends to inhibit activity in the DMN, and vice versa. The TPN is important for problem solving, focusing of attention, making decisions, and control of action. The DMN plays a central role in emotional self-awareness, social cognition, and ethical decision making. It is also strongly linked to creativity and openness to new ideas. Because activation of the TPN tends to suppress activity in the DMN, an over-emphasis on task-oriented leadership may prove deleterious to social and emotional aspects of leadership. Similarly, an overemphasis on the DMN would result in difficulty focusing attention, making decisions, and solving known problems. In this paper, we will review major streams of theory and research on leadership roles in the context of recent findings from neuroscience and psychology. We conclude by suggesting that emerging research challenges the assumption that role differentiation is both natural and necessary, in particular when openness to new ideas, people, emotions, and ethical concerns are important to success.

## INTRODUCTION

The emergence of two distinct leadership roles, the task leader and the socio-emotional leader, has been documented in the leadership literature since the 1950s (Bales, 1958). The separation of these roles has been seen as pragmatic in applications and is accepted as a finding in behavioral research. However, in practical applications, it leaves an organization with a challenge that can detract from leadership development, succession planning, and organizational flexibility. In this article, we will show that the division between these two types of leadership roles lies far deeper than has traditionally been thought. Recent research in neuroscience suggests that the division between task-oriented and socio-emotional leadership roles derives from a fundamental feature of our neurobiology: an antagonistic relationship between two large-scale cortical networks that is present in every individual.

Neural activity in the task-positive network (TPN) tends to inhibit activity in the default mode network (DMN; Raichle et al., 2001; Fransson, 2005; Raichle and Snyder, 2007; Buckner et al., 2008), and vice versa (Uddin et al., 2009; Jack et al., 2012). The TPN is activated during a broad range of non-social tasks (Fox et al., 2005; Buckner et al., 2008; Uddin et al., 2009; Andrews-Hanna, 2012), and is thought to be important for problem solving, focusing of attention, making decisions, and control of action – in other words for getting things done. However, activation of the TPN also has a deleterious effect on other cognitive functions that are essential to leadership: it suppresses activity in the DMN.

The DMN plays a central role in emotional self-awareness (Ochsner et al., 2005; Schilbach et al., 2008), social cognition (Schilbach et al., 2008; Jack et al., 2012; Mars et al., 2012), and ethical decision making (Koenigs et al., 2007; Bzdok et al., 2012; Jack et al., in press). It is also strongly linked to creativity and insightful problem solving (Subramaniam et al., 2009; Takeuchi et al., 2011). The antagonistic relationship between the TPN and DMN creates a fundamental neural constraint on cognition that is highly relevant to the different roles and capabilities that effective leaders must astutely juggle and deploy. An important consequence of this constraint is that an over-emphasis on task-oriented leadership can prove deleterious to an organization: in particular when openness to new ideas, people, emotions, and ethical concerns are important to success. On the other hand, the over emphasis on relationship oriented leadership may prove deleterious to focus and the execution of clearly defined goals.

We are not the first to note the striking correspondence between the functions of brain networks and leadership. In a recent book, social neuroscientist Matthew Lieberman distinguishes between the social brain and the business brain and does note the antagonistic relationship between the TPN and the DMN (Lieberman, 2013, p. 257). In this paper, we extend Lieberman’s preliminary work by providing a more nuanced account of the antagonistic relationship between the TPN and the DMN and their corresponding task and relationship leadership roles. We then extend Lieberman’s work by theorizing strategies that will allow leaders to effectively navigate this fundamental cognitive constraint.

## OPPOSING NEURAL DOMAINS

When discussing the functional anatomy of the brain, it is important to appreciate that the literature is less clear than is often acknowledged. The cognitive characterization of the tension between the DMN and the TPN that guides this inquiry is different in important respects from the view that is frequently stated as accepted and uncontroversial (including in the context of leadership, e.g., Waytz and Mason, 2013). Nonetheless, as we will briefly review, the characterization we offer is better supported by the scientific evidence. A number of inconsistencies in the literature facilitate misunderstanding and over-confidence: first, anatomical labels are not always used consistently and sometimes fail to distinguish areas which careful evidence reveals have quite distinct functional roles (Kubit and Jack, 2013). Second, researchers working with different types of cognitive tasks often form quite different views about the primary functional role of a region or a set of regions (i.e., a network). In particular, it is now well acknowledged that researchers have not always been careful to critically evaluate the evidence supporting their inferences about the functional role of brain areas, and sometimes fail to consider evidence about function that derives from a broader view of the literature (Henson, 2006; Poldrack, 2011). Third, networks can be defined according to a number of different criteria.

The issue of how networks can be defined is important to clarify for our purposes. Networks of regions are most often defined (and given a label denoting a provisional functional role) either because: (1) they are frequently found to be activated by a class of cognitive tasks (Corbetta et al., 1998; Duncan and Owen, 2000; Van Overwalle, 2009); or (2) the regions demonstrate strong positive resting state connectivity with each other – a finding which is commonly interpreted as indicating a degree of functional coherence amongst the regions in the network (Fox et al., 2005; Cohen et al., 2008; Vincent et al., 2008; Van Dijk et al., 2010; Yeo et al., 2011). These two criteria are thought to be complementary and broadly consistent with each other, although they do not always yield identical results (Laird et al., 2013).

A third quite different principle that can be used to define a network is through the tendency of a set of regions to be deactivated (i.e., less active than when the participant is at rest) by a class of cognitive tasks (Shulman et al., 1997b). This is how the DMN was originally defined (Raichle et al., 2001). Regions involved in the DMN have also be defined: (a) on the basis of positive resting connectivity between regions in the network, and (b) on the basis of negative correlation (“anti-correlation”) with other regions as revealed by resting functional connectivity. These three ways of defining the DMN (deactivation, positive correlation, and anti-correlation) are broadly consistent and regarded as complementary (Fox et al., 2005; Raichle and Snyder, 2007; Buckner et al., 2008; Uddin et al., 2009; Andrews-Hanna, 2012).

**FIGURE 1:**
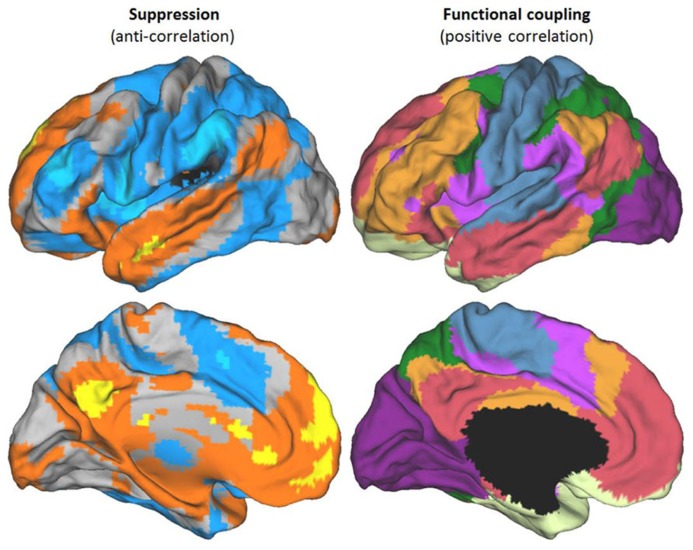
**Different criteria for defining brain networks.** Functional networks shown on the left hemisphere of the PALS atlas (*top* – lateral view above, *bottom* – medial view). Left panels show anti-correlated networks derived from resting state data. The TPN is shown in blue, the DMN in orange/yellow. Darker colors correspond to anti-correlated networks using a global (whole brain) regressor, brighter colors without a global regressor (i.e., most strongly anticorrelated areas). Maps derived from data and methods described in Jack et al. (2012). Right panels show the seven network stable solution derived by Yeo et al. (2011) on the basis of positive correlations in resting state data. Area shown in black corresponds to a cross-section through sub-cortical structures, not mapped by Yeo et al. (2011).

**FIGURE 2:**
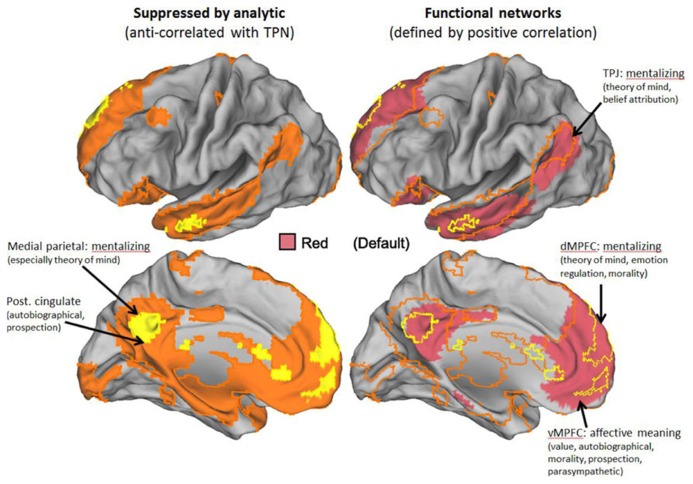
**The default mode network (DMN).** Strong overlap in the DMN representation based on anti-correlations and positive correlations in resting state data. Left panels show just the DMN derived from anti-correlations in orange/yellow. Right panels show networks derived by Yeo et al. (2011) with substantial overlap. Borders of anti-correlated regions are carried over from the left panels. Key to Yeo et al. (2011) networks is shown in the middle of the figure. Labels denote best current understanding of the primary functions of key parts of the DMN.

**FIGURE 3:**
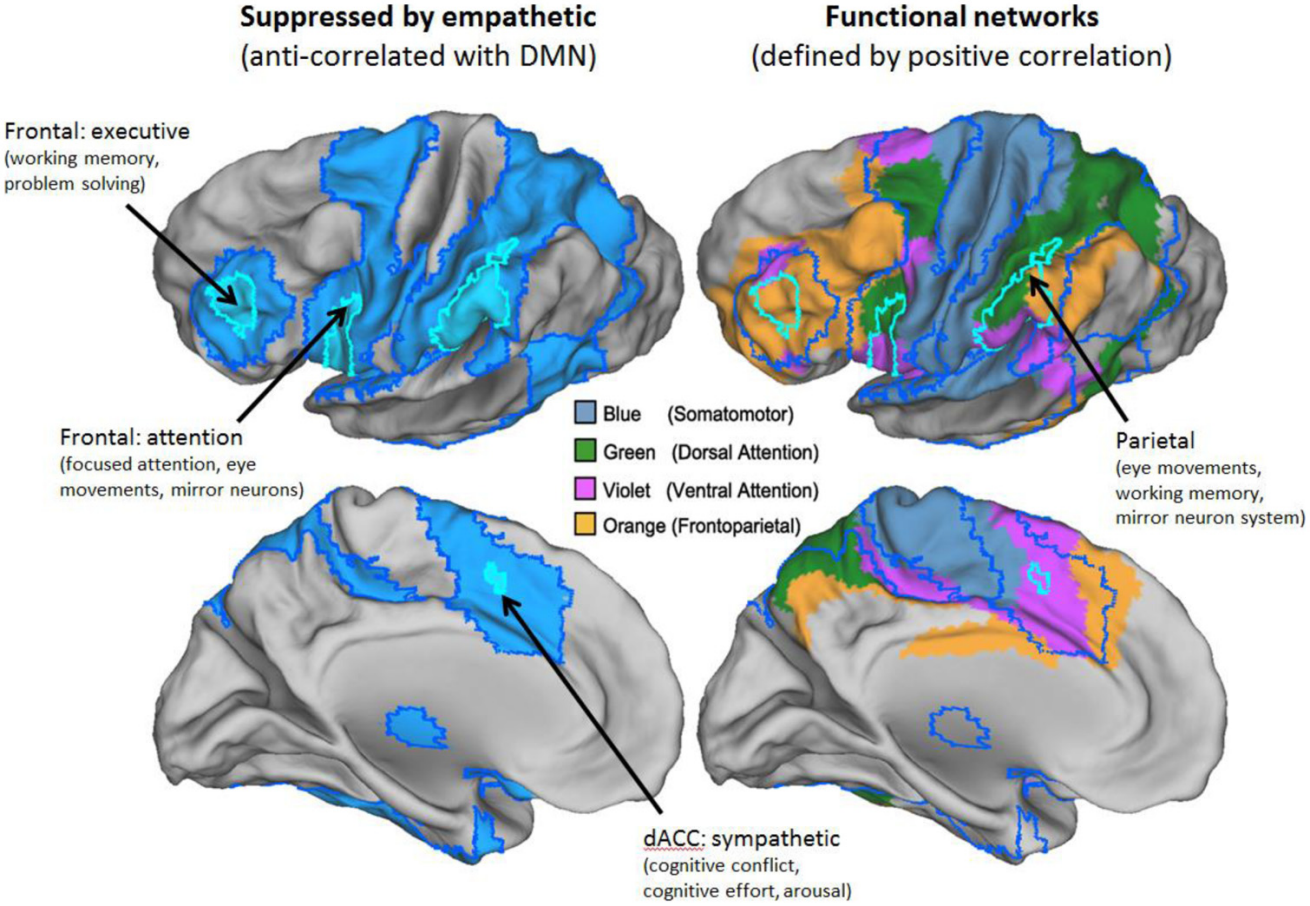
**The task-positive network (TPN).** The TPN representation based on anti-correlations corresponds to multiple networks defined by positive correlations. Left panels show just the TPN derived from anti-correlations in blue. Right panels show networks derived by Yeo et al. (2011) with substantial overlap. Borders of anti-correlated regions are carried over from the left panels. Key to Yeo et al. (2011) networks is shown in the middle of the figure. Labels denote best current understanding of the primary functions of key parts of the TPN.

The definition we take as primary for both the DMN and the TPN is their anti-correlation. This follows the original definition of these networks (Fox et al., 2005). An illustration of the two networks, defined in this way, can be seen in **Figure 1**. **Figure 1** also illustrates a recent state-of-the-art division of the entire cortex into seven networks based on functional coupling between regions, i.e., positive resting connectivity (Yeo et al., 2011). The mapping between Yeo et al.’s (2011) positive connectivity maps and the anti-correlated networks is illustrated in greater detail in **Figures 2** and **3**, for the DMN and TPN, respectively. This reveals the need for some revision of early characterizations of the anti-correlated networks. The broad characterization of the DMN has remained quite stable, and is reasonably consistent using positive and negative correlation criteria (**Figure 2**). However, this is not the case for the TPN (**Figure 3**). It was originally thought (Fox et al., 2005) that the DMN was anti-correlated primarily with the dorsal attention network (Corbetta et al., 1998). However, subsequent work using more data-driven methods (Fox et al., 2005; Chang and Glover, 2009; Chai et al., 2012; Jack et al., 2012) has revealed that the TPN overlaps parts of both the dorsal attention network and the fronto-parietal control network (Vincent et al., 2008). **Figure 3** also illustrates clear overlap between the TPN and the resting state network which Yeo et al. (2011) identify with the ventral attention network (Fox et al., 2006). The ventral attention network is recruited during a variety of demanding attention tasks, however, its precise functional characterization is still subject to debate. It has been characterized as involved primarily in the reorienting of attention, however, more recent analysis suggests that the network as identified by Yeo et al. (2011) can be better characterized as being involved in detecting and responding to task-relevant stimuli (Kubit and Jack, 2013). Hence, particularly for the TPN, it is important to note there is inconsistency between the criteria for defining networks. It appears that DMN is in tension with a set of regions (i.e., the TPN) that lie within a number of different cortical networks that can be distinguished and defined by distinct profiles of positive functional coupling.

### THE DEFAULT MODE NETWORK

Until quite recently, the function of the DMN was regarded as largely mysterious. In a comprehensive review, Buckner et al. (2008) noted that “a unique challenge for understanding the functions of the brain’s default network is that the system is most active in passive settings and during tasks that direct attention away from external stimuli.” While these and other researchers acknowledged the difficulty in definitively identifying a function for the DMN, nonetheless a consensus view began to emerge: that the tension between the TPN and DMN could be best explained by a distinction between two kinds of attention, namely externally vs. internally directed cognitive processing. This view was encouraged by some influential early work (Gusnard et al., 2001), by the mistaken view that the DMN was anti-correlated with regions whose primary function was external attention (Fox et al., 2005), and by the paucity of evidence that any externally oriented task could produce activation of the DMN above resting levels (but see Iacoboni et al., 2004).

It is clear that the DMN is reliably engaged by some tasks that involve attention to internal stimuli. Notable examples include: autobiographical recall and prospection (imagining future events; Buckner and Carroll, 2007; Spreng et al., 2009; Spreng and Grady, 2010), self-related processing (Mitchell et al., 2006), and emotion self-regulation (Ochsner et al., 2005). However, the characterization of the DMN as being primarily involved in internal processing glosses over other highly reliable findings which associate DMN activation with externally oriented tasks. These types of externally oriented tasks fall into two broad categories, which correspond to a fractionation of the DMN which can be observed both through meta-analysis of activation findings (Andrews-Hanna et al., 2010) and by resting connectivity analysis (Uddin et al., 2009). More dorsal parts of the midline structures of the DMN, medial parietal and dorsal medial prefrontal cortex (dmPFC) are reliably associated with thinking about the mental states of others, including both their emotional and cognitive (e.g., belief) states (Amodio and Frith, 2006; Schilbach et al., 2008; Van Overwalle, 2009). In contrast, ventral medial prefrontal cortex (vmPFC) is associated with representing the value of external objects (Grabenhorst and Rolls, 2011). Moral decision making tasks, which are again predominantly external in focus, are associated with activity in both dorsal and ventral parts of the DMN (Greene et al., 2004; Bzdok et al., 2012; Koenigs et al., 2012; Jack, in press). Since these findings appear to be inconsistent with the commonly held view that the tension between the TPN and DMN reflects a tension between external and internal attention, we will temporarily set aside that view and return to it in a later section. Instead, the characterization of the DMN we offer here will be guided by a broad view of tasks that are positively associated with DMN engagement.

The DMN may be seen as having two primary circuits. The first is the dorsal portions of the midline structures and the right temporo-parietal junction (rTPJ), which are most clearly associated with mentalizing, that is, thinking about our own and other’s mental states (Ochsner et al., 2005; Amodio and Frith, 2006; Schilbach et al., 2008; Van Overwalle, 2009). It is important to note that the anatomy of this mentalizing circuit is distinct from other regions that make distinct contributions to social cognition. These include: a set of regions primarily involved in perceptual processing of social stimuli such as faces and bodies (Wiggett et al., 2009) and the mirror neuron network, which is involved in both executing and observing actions, and thought to underlie our ability to mimic the actions of others. In contrast, the mentalizing system is thought to be involved not in emotional contagion, but in the cognitive representation of emotion (Lindquist et al., 2012).

The second of the primary DMN circuits is the more ventral portions of the midline structures, which are associated with self-related processing, autobiographical memory and prospection, cognitive representation of emotion, representation of value/reward, emotion self-regulation, and autonomic processing. We endorse the characterization of these processes offered by a recent review (Roy et al., 2012), which concludes: “The vmPFC is not necessary for affective responses per se, but is critical when affective responses are shaped by conceptual information about specific outcomes. The vmPFC thus functions as a hub that links concepts with brainstem systems capable of coordinating organism-wide emotional behavior, a process we describe in terms of the generation of affective meaning.” The severe consequence of poor function in this circuit has long been known through the highly influential work of Damasio (1994) looking at patients with vmPFC damage. These patients exhibit severely impaired social, moral, and decision making behavior, cannot hold down a job, and tend to be shunned by family members, even though they often have high IQs (Anderson et al., 1999). More recent work with moral decision making tasks indicates that they also tend to favor a course of action, which appears to promote the best overall outcome, even when that involves denying individual rights and directly harming others. In other words, they are more utilitarian in their thinking (Koenigs et al., 2007).

With regard to leadership roles, the DMN is the basis for relational roles in which the leader makes sense of their own and other people’s emotions and helps to construct a sense of purpose or vision for the group. Given that both subsystems of the DMN are typically deactivated by tasks that activate the TPN, it is troubling to imagine the potential consequences of placing too strong an emphasis on adopting leadership roles that recruit the TPN.

### THE TASK-POSITIVE NETWORK

The broadly defined functions of the TPN are agreed in the literature, although some debate persists about the best way to characterize the function of the finer grained functionally coupled networks which it overlaps (Kubit and Jack, 2013). The TPN is activated, and the DMN deactivated, by wide variety of non-social tasks including those involving focused attention, working memory, language, logical reasoning, mathematical reasoning, and causal/mechanical reasoning (Shulman et al., 1997a,b; Duncan and Owen, 2000; Fox et al., 2005; Owen et al., 2005; Van Overwalle, 2011). While the TPN includes some regions of the brain associated with social processes such as the mirror neuron network, it is distinct from the mentalizing network of the DMN both in its location and in the types of tasks that activate it (Van Overwalle and Baetens, 2009; Van Overwalle, 2011; Jack et al., 2012). The TPN includes parts of the dorsal attention system (Fox et al., 2005), the fronto-parietal control network (Vincent et al., 2008), and the ventral attention network (Fox et al., 2006; Kubit and Jack, 2013). Using more relaxed criteria, it can also be seen to overlap the somatomotor network (**Figure 3**). These networks are broadly associated with focus on, and execution of, well-defined tasks that are non-social in nature. Jack (in press) defines the TPN as “analytical–empirical–critical reasoning, such as mechanical reasoning.” Given our current understanding of the TPN, leadership roles associated with this network are those focused on financial planning, metrics, forecasting, problem solving, as well as strategic social engagement for the purpose of task achievement.

### THE OPPOSING DOMAINS HYPOTHESIS

As discussed, it is generally accepted in the cognitive neuroscience literature that the DMN and the TPN are anti-correlated (Greicius et al., 2003; Fox et al., 2005; Fransson, 2005; Golland et al., 2007; Tian et al., 2007; Buckner et al., 2008; Jack et al., 2013b). It is also broadly agreed that a variety of cognitively demanding non-social tasks tend to activate the TPN and deactivate the DMN. There has been less agreement about how best to cognitively characterize the tension between these networks.

The opposing domains hypothesis predicts that activation of the DMN or the TPN is a result of the type of thinking a person engages in to order to complete a given task, regardless of whether the task is externally or internally oriented (e.g., relates to perceived stimuli vs. to information recalled from memory). Personality factors play a role in the deployment of these networks, particularly for tasks where the most productive strategy is unclear or ambiguous. Nonetheless, it is thought that all neurotypical individuals are capable of flexibly deploying these networks, hence tasks which have a clear affordance for one type of processing over the other will tend to engage the relevant network. When a person engages in a cognitively demanding non-social task, they will tend to define their role as having an analytic focus. In this circumstance, both the opposing domains hypothesis and other accounts predict the TPN will tend to be activated and the DMN deactivated. When a person engages in a task that involves mentalizing and/or thinking about affective meaning, and as a result, defines their role as social and/or relational, the opposing domains hypothesis predicts the DMN will be activated and the TPN deactivated. This prediction, which entails that an individual’s analytic abilities are suppressed when they are empathically engaged with people, is unique to the opposing domains account.

Jack et al. (2012) used functional magnetic resonance imaging (fMRI) to record brain activity when participants were engaged in social vs. mechanical/analytic tasks. The social tasks required participants to answer questions about the beliefs and attitudes of the characters in emotionally and morally laden text passages or video clips. The mechanical tasks required participants to complete science puzzles, presented either as written passages, or as video clips taken from the Video Encyclopedia of Physics. A rest condition was included, in which participants were only asked to stare at a fixation point, for the purpose of establishing a resting baseline against which both activations and deactivations could be observed.

The findings showed that the neural activation during the social tasks, specifically the activation of the rTPJ, medial parietal/posterior cingulate and the medial prefrontal cortex, was accompanied by the deactivation of the neural networks responsible for mechanical reasoning, specifically, the superior frontal sulcus, lateral prefrontal cortex, and the intraparietal sulcus. Controls were put in place for task and perceptual demands to rule out the alternate hypothesis that the TPN vs. DMN dichotomy can be best accounted for by internal vs. external attention.

Jack et al. (2012) conclude that the anti-correlation between the TPN and DMN “reflects a powerful human tendency to differentiate between conscious persons and inanimate objects in both our attitudes and modes of interaction” (p. 396). Further work (Jack et al., 2013b) looking at humanizing vs. objectifying (where people tend to be viewed as objects) narratives about people provides additional support for the view that these networks are not driven by the surface characteristics of the stimuli, but rather reflect flexible cognitive stances which can be deployed depending on the attitudes and role adopted by the individual. The implications of these findings for leadership and organizational behavior are vast. The duality of task and relationship; inanimate and animate; and social and non-social can be found in the personality, motivation, group dynamics, socialization, conflict, trust, decision making, mindfulness, and moral reasoning literatures.

A further wrinkle to this account is important to note. While it is very well documented that the TPN and DMN tend to be antagonistic, both at rest and during the performance of tasks, the dichotomy between these networks is not absolute. They can be simultaneously activated and work in cooperation with each other (Fornito et al., 2012; Meyer et al., 2012). Both theory and observation indicates that this occurs when participants are engaged in a type of social reasoning that is highly instrumental, and lacking in genuine empathy. For instance, this pattern is seen more in individuals who are prone to Machiavellian thinking (Bagozzi et al., 2013). It is also the neural signature that is seen when people are animalistically dehumanized, rather than objectified (Jack et al., 2013b).

This recruitment of the TPN during social cognition is likely due to a person thinking critically, strategically, or mechanically about people. Hence, it does not appear to be the case that we can be both genuinely empathetic and analytic at the same time. Instead, our view is that when these networks work co-operatively with each other (Fornito et al., 2012), they realize a different type of cognitive processing from either genuine empathy or pure analytic reasoning. When we engage the analytic network alongside the DMN, this corresponds to a mode of social interaction that is alienating to others. This is reflected in everyday language. When we say that people are being “manipulative” or “calculating,” the metaphor may make sense because they are using TPN brain regions involved in fine motor control and/or mathematical reasoning to operate on social representations (Meyer et al., 2012). Although this cognitive mode involves a sense of social or emotional distance, it is clearly useful to think “strategically” or “politically” in this way on occasion. This is reflected in the observation that strategic social reasoning plays a crucial role in leadership research. For example, Boyatzis and Goleman (2007) make the distinction between emotional intelligence (EI) and social intelligence (SI). EI is defined as the ability of a person to recognize, understand, and use emotional information about oneself or others (Boyatzis, 2006). We expect these competencies will tend to recruit both the DMN and the TPN in tandem. By contrast, SI competencies are non-task focused, for example, acting with compassion, and would therefore primarily recruit the DMN (Boyatzis and Goleman, 2007). Interestingly, Boyatzis et al. (2006) predicted that SI competencies are linked to the parasympathetic nervous system, while focusing on tasks and instrumental use of EI is linked to the sympathetic nervous system. This would suggest that EI competencies would relate to the TPN. Research is currently underway to explore what overlap exists between the divisions of the autonomic nervous system and the TPN and DMN. While the evidence is far from conclusive, early findings based on recent meta-analyses (see, for example, Beissner et al., 2013) indicate that the TPN is more closely linked to the sympathetic nervous system while the DMN is closer to the parasympathetic nervous system. The labels in **Figures 2** and **3** indicate regions which show the closest correspondence between the DMN and TPN and regions implicated in autonomic nervous function (Eisenberger and Cole, 2012).

## OPPOSING ROLES OF LEADERSHIP

### LEADERSHIP ROLES

A role is a constellation of behavior and expectations enacted in a social situation. Mead (1934) claimed that roles were embedded in the situation and therefore were constantly emerging and evolving. As a sociologist and helping to formulate the interactionist theory of roles, Mead (1934) emphasized the expectations of others in the forming and reforming of roles. In this way, roles are distinct from style. Leadership style describes a dispositional comportment of a person as a leader that can vary by situation and demand. Leadership style contributes to role enactment as Biddle (1986) explained from a functionalist theory of roles.

In leadership, a role provides a guide to one’s behavior and helps new role occupants become socialized into how they could or should act in a way consistent with the norms and culture of an organization. A leadership role of a person emerges in response to the expectations of others around the leader, especially the followers, and the leader’s own style/s, competencies, and values (Boyatzis, 1982). This process of role emergence as a result of the interaction between expectations and behavior has been explained by cognitive role theory (LeMay, 1999).

There appears to be two distinct components of the enactment of leadership roles that parallel the distinction between the TPN and the DMN: task achievement and relationship development. We will place the evolution of this duality of leadership roles in historical context.

### FROM TAYLOR TO BASS APPROACHES TO LEADERSHIP ROLES

The centrality of tasks to a leader’s role was evident in Cowley’s (1928) definition of a leader: “an individual who is moving in a particular direction and who succeeds in inducing others to follow him…A leader then, is a person who is going somewhere, who has a motive, who has a program.” (p. 145–146). In short, without a task, leaders do not exist.

Fredrick Taylor’s scientific management movement emphasized the task aspect of leadership. Followers were viewed as “machines” to be used efficiently by the leader. In other words, by treating humans as inanimate objects subject to analytical analysis, the role of a leader in the scientific management era was largely reliant on recruitment of the TPN rather than the DMN (Jack et al., 2013b) and therefore discouraged the leader from fostering more meaningful social–emotional connections with followers. The introduction of the “human” side of leadership in the late 1930s gave rise to a multitude of leadership frameworks that reflected the scientific management (task) – human relations (relationship) duality. These frameworks include the autocratic (task)-democratic (relationship) continuum (Tannenbaum and Schmidt, 1958), the Ohio State Leadership Studies on structure (task) and relationship (Shartle, 1950; Fleishman, 1953; Halpin and Winer, 1957), and the Michigan Leadership Studies (Katz et al., 1950) of a production orientation and an employee orientation.

### ATTEMPTING TO INTEGRATE TASK AND RELATIONSHIP ROLES FROM THE 1950s TO THE 1970s

When symbolic interaction theory transferred from social to organizational psychology, leadership theory changed to require *both* relationship building and task attainment. This development parallels the duality of the neural opposing domains: relational leadership competencies are facilitated by the DMN, while the task competencies are facilitated by the TPN. Bales (1958) was instrumental in this theoretical shift in the leadership literature with his claim that a task-oriented group requires two types of leaders to maximize effectiveness; one leader to attend to the task functions of the group (instrumental leader) and another to attend to the emotional needs of the group (expressive leader). The suggestion that two leaders are required in a group stood in stark contrast to the idea that all leadership roles could be carried out by one individual. Given that we now know that the neural networks underlying these two roles are antagonistic, having two leaders in a group presents what initially appears to be a reasonable strategy for relieving the task–relationship tension.

By the 1960s, a consensus began to form that building relationships with followers was a fundamental component of leadership and a critical ingredient in task achievement. Leadership theorists become interested in developing strategies that would enable leaders to integrate these two fundamental leadership roles. The early Ohio State Leadership Studies (Fleishman, 1953) confirmed the conceptual distinction between task-oriented and relationship-oriented leadership and developed a measurement tool for each. Following from this work, task-oriented leadership has been identified as being concerned with production (Blake and Mouton, 1964), goal achievement (Cartwright and Zander, 1960; Bowers and Seashore, 1966), labor allocation, enforcement of sanctions (McGregor, 1960), and creating a context and structure for followers (Shartle, 1950; Fleishman, 1953; Halpin and Winer, 1957). At the individual level, task-oriented leaders were said to have a high need for achievement (McClelland, 1961; Wofford, 1970), to be achievement oriented (Indvik, 1986), and to be cold and aloof to signal their preference for psychological distance from followers (Blau and Scott, 1962). Interestingly, these descriptions are not only consistent with the role of the TPN discussed earlier, but also allude to the suppression of the DMN – in particular, they fit well with our observation that the socially distancing effect of dehumanizing is associated with a shift from DMN to TPN engagement (Jack et al., 2013b).

Conversely, relationship-oriented leaders were described as being focused on follower well-being (Hemphill, 1950), concerned with developing and maintaining relationships (Hersey and Blanchard, 1969), to be more democratic as opposed to autocratic (Bass, 1990), and as placing value on friendships, open communication, and mutual trust. These psychological features fit a profile of DMN activation and TPN deactivation – the neural signature associated with thinking about the experiences of others (Jack et al., 2012) and humanizing (Jack et al., 2013b). Individuals who are humanized experience positive emotions, whereas those who are dehumanized experience negative emotions (Bastian and Haslam, 2011). Correspondingly, research has shown that relationship-oriented leaders are associated with higher job satisfaction and lower turnover in organizations (Bass, 1990; Yukl, 2006).

Yukl (1999) provides the most recent taxonomy of task and relation oriented leader roles. He also distinguished a third dimension in his taxonomy, labeled “change orientation” which includes a mix of task and relationship oriented roles. However, in critiquing other’s work, Yukl (1999, 2008) notes the limitations of the task–relationships distinction, specifically, the inconsistent empirical results and the lack of evidence that it is indeed possible to be high in both task and relationship competencies and that being so is related to leadership effectiveness (Yukl, 1999). Research conducted since adds support to Yukl’s critique; specifically, Kaiser and Kaplan (unpublished, as cited in Bass and Bass, 2008) found that in a sample of managers from a consulting firm, 46% were highly task oriented, 19% were highly relationship oriented, and only 6% showed flexibility between the two dimensions. The remaining 29% were low in both task and relationship orientation.

The distinction between task-oriented roles vs. relationship-oriented roles presented in Yukl’s taxonomy appears to closely parallel the distinction made between social and mechanical tasks used to test the opposing domains hypothesis (Jack et al., 2012). Specifically, the task-oriented tasks are, by and large, mechanical and analytical in nature and would therefore activate the leader’s TPN, while the relationship oriented tasks are generally social in nature and would therefore activate the leader’s DMN.

The anti-correlation between these two networks suggests that when a leader is focused on a task-oriented role, their ability and desire to attend to the relationship needs of their followers is diminished. This is not to say that leaders do not have the ability to be both highly analytical and to build effective relationships. In the absence of any task, it is known that the human brain naturally cycles between activation of the TPN and activation of the DMN, activating each several times in a period of a minute (Fox et al., 2005). Hence, it is clearly possible to switch between networks and possibly roles.

However, the evidence from neuroscience does suggest that leaders cannot simultaneously attend to these distinct roles, hence conflicts are likely to arise if leaders get “stuck” in one role, which decreases their ability to switch between the two networks, resulting in the prolonged suppression of one of the networks and associated roles. This trade-off has been noted by a number of scholars. Most recently Yukl (2008) notes that “efforts to improve one performance determinant may have an adverse effect on another performance determinant…when leaders are preoccupied with responding to external threats (task), there is less time for people-oriented concerns such as being supportive and developing member skills” (pp. 711–714). The opposing domains hypothesis provides an alternative explanation for why this trade-off occurs. Rather than, or at least in addition to, a simple lack of time and overall cognitive resources, the opposing domains hypothesis suggests that the suppression of the opposing neural network further exacerbates the “adverse effect on another performance determinant.”

The antagonistic relationship between the TPN and DMN may also help us to understand why the type of role a leader adopts moderates the relationship between leadership role (task vs. relationship), task performance and leadership effectiveness respectively. Specifically, Burke (1965) found that a group’s performance of a coding task was completed more effectively under a production-oriented (task) leader while the shared decision-making (task) was carried out more effectively under a relationship-oriented leader. The opposing domains hypothesis offers two explanations for this finding – the first related to the ability of the followers to perform the task, the second related to the leadership role that the followers’ perceived to be effective.

First, followers performed more effectively on the analytical task when the leadership role matched the neural network activated as a result of the analytical nature of the task (the TPN). When a relationship-oriented leadership role was used, the leader’s behavior and focus on relationships may have activated the DMN in followers, which we now know to be associated with lapses in attention and performance errors (McKiernan et al., 2003; Mason et al., 2007; Fassbender et al., 2009; Pyka et al., 2009), hence the lower performance on the analytical task. Conversely, the shared decision task, which required interpersonal interaction and therefore would have activated the DMN, was performed more effectively under a relationship oriented leader. A relationship-oriented leader in this task would have allowed for the follower’s DMN to be dominant, which is the network required to perform this task effectively. A task-oriented leader would have activated the follower’s TPN, which would suppress the DMN resulting in a reduction of human and ethical insight (Jack et al., 2013b, in press) and consequently lower task performance.

Second, when completing the coding tasks the followers were predominately engaging their TPN, hence the task-oriented leadership role may have been perceived by followers to be more effective because it was more psychologically resonant. Similarly the relationship-oriented leadership role may have been preferred by followers engaged in the moral decision task because of the resonant emphasis on DMN engagement for both the task and the leadership role.

Further evidence of the interaction between task type and follower preference for a task vs. relationship-oriented leader is offered by Weed et al. (1976). This study found that the only type of task that was a significant moderator between leadership role and task performance was the difficult–ambiguous task. While this study was focused on the difficulty and ambiguity of the task rather than the mechanical or social nature of the task, the authors note that the difficult–ambiguous task was the only task that involved ethical/moral problems and therefore “the significant findings on this task may be partially attributable to the types of skills required to deal with these problems” (p. 65).

Finally, Hersey and Blanchard (1969) developed the Tri-Dimensional Leadership Model that sought to link task and relationship oriented leadership roles to follower perceptions of leadership effectiveness. The Tri-Dimensional Leadership Model suggests that leaders who are highly task oriented will be perceived as “effective” because they know what they want and are able to impose this to accomplish a task without causing resentment. A highly task-oriented leader will be perceived as ineffective when followers’ perceive the leader has no confidence in others, is unpleasant, or only shows interest in short-run output. In other words, a highly task-oriented leader will only be seen as effective if they are aware of, and attend to, relationships – rather than dehumanizing their followers – while accomplishing the task.

Similarly, a leader with a high relationship-orientation will be seen as effective by followers when they perceive the leader has implicit trust in them and is primarily concerned with developing their talents – behaviors associated with humanizing and the DMN. They will be seen as ineffective when followers perceive the leader to be passive and showing little care about the task at hand – behaviors associated sustained activity of the DMN that suppresses the TPN (Mason et al., 2007; Jack et al., 2012). These findings extend the hypotheses derived from the Burke (1965) study with the suggestion that regardless of a leader’s preferred role (task-oriented or relationship oriented), the leader must be able to switch fluidly between the opposing neural networks (the TPN and the DMN) in order to be perceived as effective by their followers. Given this, it seems that rather than identifying the individual difference variables and situational variables that are associated with each style in isolation, it may be more important to identify variables that correspond to the ability of a leader to switch between the TPN and the DMN in order to maximize leader effectiveness.

## DEALING WITH THE DIALECTICAL TENSION

The previous section has shown that the distinction between task and relationship roles is evident throughout the leadership literature. In this section, we explore potential strategies to resolve, or at least minimize the consequences of the tension that leaders face in developing their roles to attend to task requirements and attending to relationships as a result of the antagonism between the TPN and the DMN.

### NEURAL DISPOSITION: MATCHING THE LEADER TO THE SITUATION

As with hormonal disposition, there is some evidence to suggest that humans have a natural disposition toward either analytical–mechanical reasoning and therefore the TPN or social–relational reasoning and therefore the DMN (Jack et al., 2012). While neural disposition is a relatively nascent area, hormonal dispositions are well documented in the literature (see Insel, 1997; Schulkin, 1999). For example, people with higher unconscious power motives are known to have higher resting levels of epinephrine secretion, while people with a higher unconscious Need for Achievement appear to have higher resting levels of vasopressin secretion (Boyatzis and Sala, 2004; Boyatzis et al., 2006). At the behavioral level, neural and hormonal dispositions may underlie certain personality characteristics, learning styles, and perhaps preferred leadership roles (Boyatzis and Sala, 2004).

Considering the opposing domains model, one way to address the tension between the task and relationship leadership roles could be to match a leader’s predisposition toward either task or relationship to the type of task they perceive as their primary role and responsibility. In many ways, Bales’ (1958) suggestion of the need for two leaders was an attempt to do this. The utility of matching a leader’s natural inclination toward either a task or relationship role to the situation has found some empirical support in the literature. For example, Slatter (1955) found that when task demands are high, being liked does not contribute to leadership effectiveness and social or relational skills are not highly valued, whereas in therapy groups, sensitivity training groups, and social clubs socio-emotional skills were important. However, the practical results from dividing the leadership roles have been less than ideal.

Like the old CEO (chief executive officer) and COO (chief operating officer) split, father and mother role split, CO (commanding officer) and XO (executive officer) split in the military, this leadership role differentiation is possible but appears to be less effective in the long-term. Attempts to have two people occupy co-chair, or co-CEO roles have been, at best, confusing. Usually leaders in these roles cannot sustain true status and power equalization over time. In addition, others around them may not allow it and their expectations and preferences push for more status differentiation, not less. Over time, one role dominates the other, and as a result one neural network dominates brain activation (even as an organizational norm) leading the organization down a narrow path of either problem focused decisions with little openness to new ideas or events occurring in the larger environment (like market shifts) OR an organization preoccupied with environmental changes and meeting the various desires of employees that has difficulty executing a strategy consistently over time.

Differentiating the leadership role effectively allows a leader to spend the majority of their time in one of the two neural networks and reduces the need to cycle between them. From the point of view of sustained leadership effectiveness, fluid cycling at rest between the two networks is associated with good mental health and higher IQ, whereas a lessening of the cycling between networks is associated with a variety of mental disorders (Broyd et al., 2009; Andrews-Hanna et al., 2010; Anticevic et al., 2012; Whitfield-Gabrieli and Ford, 2012). While studies of the long-term effects of privileging the engagement of one cognitive mode over the other in task performance have yet to be done, it is plausible that a more balanced approach is associated with better long-term mental health and performance, whereas over-privileging one network for sustained periods leads to mental exhaustion and burn-out – two detrimental effects that are often discussed in the leadership literature. Therefore, role differentiation, while presenting an easier short-term strategy for an organization to accomplish a balance, may prove far less productive if the individual’s role remains constant over time.

These considerations suggest a more effective approach would be to train and develop leaders so that they not only possess a high level of competency in enacting both the task and relationship leadership roles, but also have the ability to switch fluidly between them along an awareness of appropriate cues and contexts for doing so (French and Jack, in press), which also requires a perceptual facility with perceiving when each is needed or more appropriate. The actions to invoke a change of role are within a leader’s purview. For example, a leader witnessing a competitor take some of their clients or market share could see this as a need for analytic investigation – is there is pricing difference? Are there differences in transportation costs or delivery speed?

Similarly, a leader who is heavily into a task role (and TPN activation) may decide that each day he/she will coach another person to help them develop. In a within subjects study comparing a method of coaching with the positive emotional attractor (PEA; i.e., coaching with compassion) vs. the more typical method of coaching someone to fix them – to the negative emotional attractor (NEA; i.e., coaching for compliance), components of the DMN were significantly activated in the PEA condition more than the NEA condition (Jack et al., 2013a). Since this neural network allows a person to be more perceptually and cognitively open to new ideas, it may be the process needed to help people become open to switching and engaging both domains with fluidity. By deciding to coach one person each day (formally or informally over coffee or a lunch), *and* to commit to doing it about the person’s dreams, vision or values, the leader commits to switching into the relationship role at least once a day and activating the DMN in both himself/herself and the other person. Over time and practice, the switching between the task and relationship roles would likely become easier and help the leader develop more cues as to when one role is more appropriate or possible than the other. Not quite as simple as changing hats or shoes, but it could become as convenient.

### NEURAL RESOURCE EFFICIENCY

The opposing domains hypothesis could be framed as presenting a form of “trade-off” between adopting roles favoring task-related leadership activities and therefore activating the TPN and suppressing the DMN and adopting roles favoring relationship building activities and therefore activating the DMN and suppressing the TPN. In this framing, neurological activation is essentially a form of resource that leaders distribute between the task and relationship roles to attain or increase their effectiveness. When a leader expends more neural resources attending to relationships, they consequently have fewer resources to invest in the task role and vice versa. It may be possible that we can change the rate or efficiency of the leader’s neural resources, thereby minimizing the extent of activation required to successfully complete the task. Because the TPN and the DMN are antagonistic, minimizing the degree of activation in one network also serves to proportionally decrease the suppression of the opposing network (McKiernan et al., 2003; Pyka et al., 2009).

#### Task-positive network competencies

We expect that leaders with a high level of cognitive intelligence (g) will show greater ability in analytical or mechanical type tasks. They are also more likely to perceive the need for an analytic leadership role focusing on the TPN and to adopt this role in an organization. When faced with an analytical or mechanical task, we expect leaders with high levels of cognitive intelligence will require less “effort” or neurological resources to successfully complete an analytical or mechanical task than leaders with lower levels of cognitive intelligence (Graham et al., 2010). Similarly, we expect that when faced with an analytical task requiring the strategic use of emotional information, leaders with high EI will require less cognitive effort to engage followers than leaders with low EI. Leaders with higher levels of EI can access the emotional information more easily than those with low EI, even if it is in service of an analytic task. This helps to clarify that emotional labor can be in service of either a task demand or an emotional and social demand.

A leader who shows a high level of familiarity or specialized competence in a given task should also require less neurological resources than leaders with a lower level of familiarity or specialized competence. A high level of task competence will result in the leader experiencing the task with less intensity resulting in a lower level of activation in the TPN. By the work appearing more routine to a leader using an appropriate role, it may allow a leader to switch roles more easily. Holding competence, experiences, and intelligence constant, more difficult tasks will require more neurological resources than easy tasks (McKiernan et al., 2003; Mason et al., 2007; Pyka et al., 2009).

#### Default mode network competencies

In similar vein to the reasoning discussed above, when faced with a social or relational task, we expect leaders with high SI (as opposed to EI – see Boyatzis and Goleman, 2007) to require less neurological resources to successfully complete a relational task relative to those leaders with low SI. We would also expect that leaders who possess high levels of empathetic concern and compassion to be in a similar position. These leaders are also more likely to perceive the need for and adopt a relational leadership role.

### SWITCHING BETWEEN THE TPN AND DMN

While the idea that a task can be classified as either analytical or social is useful for theoretical purposes, as with most dichotomies, the distinction is rarely so clean-cut in practice. In reality, and particularly in the context of leadership, all tasks have a relational component and an analytical component. Leadership almost always requires consideration of both analytical tasks (TPN) and relationships (DMN), therefore we suspect that the greater ability a leader has to switch between these two modes of reasoning the more effective they will be as a leader. We suspect that minimizing the suppression of the opposing network will make it easier, faster, and less costly for the leader to switch between the two networks. For example, we have already argued that leaders with certain social competencies require less cognitive effort to complete a social task than those without these competencies, resulting in less activation of the DMN *and* less suppression of the TPN. This reduction in the difference or gap between the two networks should make switching between the two networks faster and less costly.

Along with the ability to switch between the two opposing networks, which we argued may require a reduction in intensity of the dominant network, we also expect the ability to appropriately time the shift from task to relationship to be important both in terms of minimizing disruption for followers and for maximizing the effectiveness of the shift. For example, knowing that activation of the DMN suppresses our analytical–mechanical reasoning abilities (Jack et al., 2012), and that activation of the DMN during analytic tasks is associated with mind-wandering and lapses of attention (Mason et al., 2007; Fassbender et al., 2009), it would appear unwise for a leader to engage followers in activities requiring social or relational reasoning at a time when it is important or urgent to maintain analytic focus. Similarly, in situations requiring social or relational reasoning, for example, during the group’s formation period, introducing task-focused activities may inhibit relationship development in the group.

The timing of the switch may also have implications for the timing of feedback. Based on the opposing domains hypothesis, we would expect that the closer the time period between action and feedback, the more congruence there should be between the type of feedback and the type of task. For example, if feedback is being given while a person is performing an analytical task, the feedback given should be analytical or technical in nature because this is the type of feedback is consistent with the neural network within which the receiver is engaged. If the leader wishes to give feedback that requires the follower to engage in social or relational reasoning, he/she would be better to wait until the receiver has disengaged from the analytical task. The same can be said for giving task-related feedback in an emotionally charged situation. Gottman et al. (2002) documented something every married or partnered person should know. When your spouse or partner is angry and yelling at you, this is not the time to reply by analyzing the situation in emotional distant terms. It does not help and in fact, as Gottman et al. (2002) document, inflames the situation.

Finally, given that the decision to switch from one cognitive domain to another requires reasoning about the emotional state of self and others, we would expect that leaders with greater DMN abilities are more likely to be able to successfully time the shifts than leaders who lack such abilities.

In sum, by using the evidence of these antagonistic neural networks and recent research on activating each of them, we can hypothesize that that most effective leadership requires a combination of three facilitating factors: (a) a decrease in the switching time (or cycle time) between these networks; (b) training people to high levels of competence in enacting the roles requiring each network, so decreasing the cognitive effort required and hence the degree of deactivation of the opposing network; and (c) training leaders to recognize and perceive contexts and cues which require a switch between modes, so they do not remain “stuck in set” and apply an ineffective cognitive strategy for the task at hand (e.g., by privileging analytic thinking when faced with an ethical decision, or intuitive thinking when faced with a logical task where creativity is not helpful).

To make this happen, we conjecture that people would have to be trained in multiple techniques that invoke a tipping point. These techniques would function somewhat like cognitive behavioral therapy, helping leaders to identify external (e.g., follower thoughts and emotional state) and internal (e.g., own thoughts and emotional state) cues and respond appropriately. In particular, people may need to develop the ability to calm the system and lower the intensity when there is a danger they are becoming “stuck in set” – whether that involves preoccupation with social/emotional concerns (over-privileging the DMN) or an overly narrow task focus (over-privileging the TPN).

This emphasis contrasts with current beliefs that to motivate we must increase energy and incentives (whether positive or negative). While that approach may preclude the dualistic swing between these two cognitive modes, and the occasionally conflicting considerations raised by each, it also presents a larger danger: losing sight of important insights, either emotional or analytic. Additionally, the ability to sense the optimal timing and context for switches may also be a critical component in understanding leadership effectiveness. For example, in the context of learning a new task, interruptions can be extremely costly, not only in terms of task outcomes (errors) but also in the ability to “pick up where you left off.” Once a person has gained a higher level of mastery, interruptions will be less costly and following the interruption, the person will be able to re-engage with the task faster.

Prior research on the intensity of emotions suggests that in order to move a person from a negative emotional state (NEA) to a positive emotional state (PEA), the intensity of the emotion must be reduced to reach a tipping point (Boyatzis, 2013). It seems reasonable to suggest that a similar principle may exist when switching between the TPN and the DMN. Boyatzis (2013) argues that when people are in the PEA they are “more perceptually open and accurate in perceptions of others” (p. 1978; see also Fredrickson and Branigan, 2005; Boyatzis, 2006), which is consistent with the work of the DMN in allowing individuals to engage in reasoning about the emotions of others. In contrast, the NEA is said to be linked to human survival, particularly to defend against threats. NEA also balances “unchecked optimism” through suppressing the DMN, which has been linked to poor investment decisions (Gibson and Sanbonmatsu, 2004) – an analytical task that would require activation of the TPN rather than the DMN. The opposing domains model suggests the NEA’s ability to balance unchecked optimism is achieved through both activating the TPN required for analytical reasoning and suppressing the DMN, which is largely responsible for the overly optimistic state.

The link between the PEA–NEA and the DMN–TPN is also reflected in Fiedler’s (1986) cognitive resources theory. Fiedler and McGuire (1987, as cited in Bass and Bass, 2008) found that under non-stressful conditions, leaders with fluid intelligence (IQ) perform better than leaders with crystallized intelligence (experience); however under stressful conditions, leaders with crystallized intelligence performed better. Cognitive resource theory posits that the reason for this distinction is that under stressful conditions, a leader with fluid intelligence will rely on intellectual solutions to a task even when such solutions are inappropriate. In other words, under stressful conditions, a leader is “stuck” in the TPN and also in the NEA due to the stress condition, which limits their ability to switch into the DMN and the PEA, which enables them to explore more creative and non-intellectual solutions. Leaders with crystallized intelligence (intelligence based on past experience and learning) are likely to experience the same situation with less intensity, thus these leaders will be: (1) closer to the NEA–PEA tipping point; and (2) more able to switch between the TPN and the DMN.

### FURTHER CONSIDERATIONS ABOUT MAPPING BRAIN NETWORKS ONTO LEADERSHIP ROLES

In this article, we have focused on mapping a duality which has long been noted at the behavioral level, between different leadership roles, onto a duality in the brain, highlighted by recent research in neuroscience. There appears to be a very promising mapping between these two domains, which suggests a fundamental neurophysiological basis for the observed duality in leadership roles. At the same time, we acknowledge that there is a considerable distance between neurophysiological observations and leadership behavior. Hence, more research is needed to firmly establish the links we highlight, and to elaborate and extend the model sketched here. Our main goal has been to highlight these links as a very promising avenue for further research. With an eye to this future research, in this section, we respond to three specific questions that naturally arise in response to our proposed mapping.

First, the DMN has been found to exist in many species (Mantini et al., 2011). Further, in humans its function serves as basic index of level of consciousness, even in non-communicative brain-damaged patients (Vanhaudenhuyse et al., 2010). Given that this network appears to have such basic functions, it may seem surprising that we are suggesting it plays a key role in effective leadership, which would appear a higher level function. However, we regard this as consistent with our account, which is based on the view that the default network originally evolved to play a basic role in visceral awareness and emotion self-regulation, and that these functions expanded and evolved so that in the human this cortex additionally supports complex representations of value and the mental states of others (Jack et al., 2012, 2013b, in press). This fits with evidence that the default network is considerably expanded in the human compared to other species, even when its size is considered relative to overall cortical volume – which is massively expanded in the human compared to other primates (Jack et al., in press; **Figure 1**).

More broadly, our review indicates that we see the default network as critically involved in self-management, in particular mindfulness, motivation, and affective meaning. We see relational aspects of leadership as an extension of these functions which arises through coupling of them with our capacity to metalize. In summary, we suggest it quite natural to view the relational aspects of effective leadership as a skillful cognitive blending of our basic capacities for emotional self-regulation and social cognition. This view sits very well with what is known about the function and evolution of the DMN, and is not contradicted by findings which indicate the DMN plays a role in more basic functions.

Second, we admit and welcome the possibility that there are likely to be additional mechanisms, beyond the DMN/TPN duality we highlight, which are critical for understanding leadership. For example, we have highlighted a duality which places two much discussed systems involved in social cognition in opposition: the mirror neuron system lies within the TPN, and hence there is a general tendency for it to be deactivated when the metalizing system of the DMN is activated, and vice-versa. The mirror neuron system is thought to play a role in “emotional contagion” (Hatfield et al., 1994) – a key mechanism used by leadership scholars to explain the transfer of emotion between leader and follower (Bono and Ilies, 2006; Johnson, 2008; see also Sy et al., 2005) and from followers to the leader (Dasborough et al., 2009). Additionally, parts of both the mirror neuron network and the DMN were activated in older executives when remembering specific moments with resonant vs. dissonant leaders in their past (Boyatzis et al., 2012). In summary, it is not our claim that the DMN/TPN dichotomy highlighted here represents an exhaustive description of the neural processes involved in effective leadership. We look forward to future research that may clarify different types of neural interaction. In particular, we acknowledge the importance of looking at ways in the DMN and TPN work cooperatively in addition to our focus here on the “default” tendency for them to work competitively (Fornito et al., 2012). Such cooperative interactions between the networks need not always be anti-social in effect, although we document evidence above that some modes of cooperation are associated with a greater sense of social distance.

Finally, the methodological concern might be raised that our analysis depends on reverse inference (Poldrack, 2011). That is, since brain imaging evidence is essentially correlational in data, it is not clear that the DMN and TPN are essential to the specified roles in effective leadership. We acknowledge this concern, which applies to all neuroimaging data. One important way to mitigate faulty inferences of this type is to conduct broad analyses of the literature in order to justify the claimed association between a specific brain area and a specific function (Poldrack, 2011). Another important step is to conduct critical tests of the hypothesized account against other accounts.

We have taken both these steps, using both meta-analysis (Jack et al., in press) and critical tests of our theory (Jack et al., 2012, 2013b) to justify our view that the DMN vs. TPN dichotomy reflects a tension between empathetic vs. analytic reasoning, as opposed to the more broadly stated view that it reflects a tension between internal vs. external attention. Nonetheless, we agree that further testing is wanted. Ideal tests would involve directly up or down-regulating one of the networks, or modifying the ability to switch between them, and then assessing the impact on naturalistic leadership behavior. As noted, there is already evidence suggesting that patients with vmPFC damage perform poorly in relational leadership roles (Anderson et al., 1999), however it would be ideal to manipulate neural processing and study effects within individuals. While this is challenging to do directly, some more indirect tests along these lines may be possible. For instance, oxytocin administration appears to up-regulate DMN function (Bethlehem et al., 2013), hence we would predict oxytocin administration should improve performance in relational leadership roles and deteriorate performance in task-oriented roles. Alternatively, it has been shown that different forms of meditation tend to either increase (focused meditation) or decrease (non-dual awareness meditation) anti-correlations between the DMN and TPN (Josipovic et al., 2012). The former should increase leadership flexibility (i.e., the ability to switch between different roles and perform well in both), the latter decrease it. Exploring these and other potential manipulations of neural processing is an important area for further research.

## CONCLUSION

The emergence of two distinct leadership roles, the task-oriented leader and the relationship-oriented leader, has been documented in the leadership literature since the 1950s (Bales, 1958). Recent discoveries in neuroscience that the TPN, which allows us to focus on problem-solving and analytic work, is antagonistic with the DMN, which allows a person to be socially engaged and open to new ideas, creates a dialectical tension that reverberates throughout the leadership role literature and raises questions as to how leaders can effectively fulfill both roles.

### RESEARCH, THEORY, AND PRACTICAL IMPLICATIONS

This paper has identified a key pattern in the leadership literature and linked this pattern to cutting edge research in the neuroscience domain. In doing so, we have raised a number of questions regarding our treatment of the task and relationship distinction in the literature to date, particularly the assumption that leaders are able to attend to both leadership roles simultaneously. Additionally, we have been able to add further explanation to some historical findings attempting to understand the interaction between task, leadership roles, and leadership effectiveness. Finally, we suggested an array of conceptual implications that might extend our current conceptualization and operationalization of leadership effectiveness.

From a practical standpoint, this paper suggests that developing a leader’s analytical and relational abilities may be an important way to offset the costly effects of the antagonistic relationship between the TPN and the DMN. Increasing a leader’s abilities in each role should facilitate the ability faster and more fluidly between the task and relationship roles by reducing the cognitive effort, and consequently the differential activation between the TPN and the DMN, required to perform effectively in each respective role. We believe that the ability to switch between these networks and corresponding leadership roles may be a key component of leadership effectiveness.

Additionally, knowing that engagement in analytical tasks inhibits our ability to engage in social or relational reasoning and vice versa may have important implications for organizations in terms of how they structure and order tasks that have analytical and relational components. For example, when giving feedback, managers should consider if the feedback is analytical or task related in nature or interpersonal in nature and time the delivery of that feedback accordingly. The same may be said for the ordering of meeting agendas and the time and structure of performance review meetings.

While this paper has focused specifically on the implications of the opposing domains hypothesis for leadership roles, we believe that the distinction and antagonistic relationship between analytical–mechanical reasoning and social reasoning exists in many other areas in the organizational behavior domain. These areas include, but are not limited to, leadership styles, conflict management, trust, and moral reasoning. For example, distinctions in the literature have been made between cognitive and relational trust (Lewis and Weigert, 1985); cognitive conflict and affective conflict (Jehn, 1997; De Dreu and Weingart, 2003); empathy and dehumanization (Haslam, 2006).

Relevant to the leadership domain specifically, further testing is needed in order to understand if individual difference variables play a role in facilitating the ability to switch between the two networks. Research is currently underway to address this question by surfacing the opposing domains at the behavioral level and linking individual difference variables and abilities to the speed at which an individual is able to switch between tasks requiring analytical and tasks requiring relational reasoning. Once we have more information on this we will be able to target these variables in leadership development training programs. Additionally, manipulation of situational characteristics within each type of task, for example, task difficulty and routineness for analytical tasks, and prior relationship quality for social tasks, will allow us to isolate the key situational variables at play. Finally, further research on the link between hormonal systems and neurological systems would allow us to understand how tipping points in hormonal systems influence neurological tipping points.

## Conflict of Interest Statement

The authors declare that the research was conducted in the absence of any commercial or financial relationships that could be construed as a potential conflict of interest.
